# The leisure basis of caring

**DOI:** 10.3389/fpsyg.2023.1067569

**Published:** 2023-04-19

**Authors:** Robert A. Stebbins

**Affiliations:** University of Calgary, Calgary, AB, Canada

**Keywords:** caring, leisure, caring activity, volunteering, compassion

## Abstract

The social science literature on caring is long on application and short on concepts about its nature. In this latter capacity leisure is seldom mentioned as a context within which people care for others. This article examines the relationship of caring and compassion as expressed during free time in leisure activities. This has never been done, with the result that many contextual and motivational properties unique to leisure have been overlooked, and consequently, have been unavailable to both the theoretic and the practical sides of the sociology and psychology of compassion. The present article attempts to demonstrate that such oversight has denied this field some useful conceptual tools.

## 1. Introduction

Caring has been defined as the process of assuming personal responsibility for others’ welfare, accomplished by acknowledging their needs and acting responsively toward them (Smith et al., 2006, p. 34). Although, in this article, discussion will center exclusively on social caring, the caring of other people, note that Oliner and Oliner (1995) broadly define the process to include both people and the natural environment. They hold further that caring is a social process; it includes both ‘attaching’ processes (bonding, empathizing, learning caring norms, assuming personal responsibility) and ‘including’ processes (diversifying one’s interaction to include those unlike oneself, networking, resolving conflict and linking the local with the global). According to Wuthnow (1991) caring is motivated, in substantial part, by compassion, or the sympathy generated by feeling another person’s suffering, which leads to an inclination to show mercy for or give aid to–care for -- that person.

In this article I examine the relationship of caring and compassion as expressed during free time in leisure activities. This, to my knowledge, has never been done, with the result that many contextual and motivational properties unique to leisure have been overlooked, and consequently, have been unavailable to both the theoretic and the practical sides of the sociology and psychology of compassion. The present article attempts to demonstrate that such oversight has denied this field some useful conceptual tools.

## 2. Three caring roles

Assuming a person is compassionate about someone else’s situation in life and therefore wants to care for that individual, what roles are open to the first? So far, I have identified three. One role is *occupational*; some workers who are compassionate about other people make a living, at least in significant part, by caring for them, often done as a professional calling (e.g., clergy, physicians, social workers). Another role, which is available outside work, is caring for other people as a *personal obligation*. Here the caring individual, fired by compassion, feels a moral duty to care for another person or class of people. Personal caring, as I will explain more fully in the next paragraph, is predominantly disagreeable; it cannot be considered leisure. Rather it is the lot of those who, though they would rather be doing something else, find themselves caring, as an example, for an ailing relative or close friend or feel morally pressured to aid the needy at home or abroad.

*Leisure caring*, our third role and the one most central to this article, refers to people engaged in uncoerced compassionate activity during free time, activity they want to do and, in either a satisfying or a fulfilling way (or both), use their abilities and resources to succeed at doing (the general definition of leisure inherent in this statement is considered more fully in Stebbins, 2005). Leisure caring is distinguished from its occupational and personal counterparts by, among other qualities, the fact that it alone is executed in free time. Still leisure caring is certainly capable of generating obligations. Be that as it may serious leisure research has demonstrated through several studies (Stebbins, 2000) that, because obligations here are agreeable, they are *defined* by committed participants as minor, as ‘minimal’. Such obligations are real, nonetheless, even while the powerful rewards of the activity significantly outweigh them and the participant has the option to quit the activity at a convenient point in the near future. More precisely serious leisure has often been found to contain some *flexible* obligation, or a relative freedom to honor commitments. This condition is generally missing in occupational caring and personal obligation.

## 3. The serious leisure perspective

A primary reason for conceptualizing caring as leisure -- when it is not occupational or personal obligation -- is that its motivational and socio-cultural (contextual) foundation differs substantially from the other two types. In general, that foundation shows people in leisure seeking, as stated in the preceding definition, satisfying or fulfilling activity that they want to do and have the abilities and resources to succeed at this. Yet leisure activities are not all cut from the same cloth, and so this broad motivational-contextual statement needs further elaboration.

This is achieved through the serious leisure perspective; a theoretic framework that synthesizes three main forms of leisure, showing, at once, their distinctive features, similarities, and interrelationships (fully explained in Stebbins, 2004/2014). Those forms are serious, casual, and project-based leisure, briefly defined as follows:

- *Serious leisure*: systematic pursuit of an amateur, hobbyist, or volunteer activity sufficiently substantial, interesting, and fulfilling for the participant to find a (leisure) career there acquiring and expressing a combination of its special skills, knowledge, and experience.
- *Casual leisure*: immediately, intrinsically rewarding, relatively short-lived pleasurable activity, requiring little or no special training to enjoy it.
- *Project-based leisure*: short-term, reasonably complicated, one-shot or occasional, though infrequent, creative undertaking carried out in free time, or time free of disagreeable obligation.

Although it was never my intention as I moved over the years from one study of free-time activity to another, my findings and theoretic musings have nevertheless evolved and coalesced into a typological map of the world of leisure. That is, so far as I can tell at present, all leisure (at least in Western society) can be classified according to one of these three forms and their several types and subtypes. More precisely the serious leisure perspective offers a classification and explanation of all leisure activity and experience, as these two are framed in the social- psychological, social, cultural, and historical contexts in which the activity and experience take place.

Additionally, each leisure activity is centered on one or more core activities, and it is here that the participant finds the most rewarding experiences possible in that activity. A *core activity* is the distinctive set of interrelated actions or steps that must be followed to achieve an outcome or product that a leisure participant finds attractive. The core activity of the larger leisure activity of which it is a part is a value in its own right, even if more strongly held for some leisure activities than others (Stebbins, 2004/2014, pp. 1–2).

Returning to serious leisure, note that it is further defined by six distinguishing qualities (Stebbins, 2004/2014, pp. 11–13), qualities found among amateurs, hobbyists, and volunteers alike. One is the occasional need to *persevere*, such as in learning how to be an effective museum guide. Yet, it is clear that positive feelings about the activity come, to some extent, from sticking with it through thick and thin, from conquering adversity. A second quality is that of finding a *career* in the serious leisure role, shaped as it is by its own special contingencies, turning points and stages of achievement or involvement. Careers in serious leisure commonly rest on a third quality: significant personal *effort* based on specially acquired *knowledge*, *training*, *experience*, or *skill*, and, indeed, all four at times. Fourth, several *durable benefits*, or broad outcomes, of serious leisure have so far been identified, mostly from research on amateurs. They are self-development, self-enrichment, self-expression, regeneration or renewal of self, feelings of accomplishment, enhancement of self-image, social interaction and belongingness, and lasting physical products of the activity (e.g., a painting, scientific article, piece of furniture). A further benefit -- self-gratification, or the combination of superficial enjoyment and deep fulfillment -- is also one of the main benefits of casual leisure, where however, the enjoyment part dominates. Of these benefits, self-fulfillment -- realizing, or the fact of having realized, to the fullest one’s gifts and character, one’s potential -- is the most powerful of all.

A fifth quality of serious leisure is the *unique ethos* that grows up around each instance of it, a central component of which is a special social world where participants can pursue their free-time interests. Unruh (1980), p. 277 developed the following definition:

A *social world* must be seen as a unit of social organization which is diffuse and amorphous in character. Generally larger than groups or organizations, social worlds are not necessarily defined by formal boundaries, membership lists, or spatial territory… A social world must be seen as an internally recognizable constellation of actors, organizations, events, and practices which have coalesced into a perceived sphere of interest and involvement for participants. Characteristically, a social world lacks a powerful centralized authority structure and is delimited by… Effective communication and not territory nor formal group membership.

The sixth quality revolves around the preceding five: participants in serious leisure tend to *identify* strongly with their chosen pursuits. In contrast, casual leisure, although hardly humiliating or despicable, is nonetheless too fleeting, mundane, and commonplace for most people to find a distinctive identity there.

## 4. Caring as a leisure activity

The compassionate person caring for someone as a leisure activity is, to be more precise, engaged in one of the three types of volunteering: *career*, *casual*, or *project-based*. The above-mentioned motivational and contextual background which explains the three leisure forms, in general, also explains these three types of volunteer activities, in particular. This background varies for all three types, however, a condition that underscores the importance of viewing care giving through the broad lens of the entire serious leisure perspective. Smith et al. (2006), pp. 239–240 define *volunteer* as someone who performs, even for a short period of time, volunteer work in either an informal or a formal setting. Moreover, consistent with the definition of volunteering, caring as volunteer activity is carried out beyond the volunteer’s family. This condition suggests that care within the family circle must be conceived of in terms other than volunteering, even if it may be held by the carer to be leisure. Treating of it as family leisure – that is, when not felt as personal obligation–would be one way of doing this.

The care-giving career volunteer meets the six qualifying characteristics of serious leisure in general. Examples of such volunteers include people who (1) work with homeless youth to facilitate their integration in mainstream society; (2) first establish and then help run a local immigrant welcoming organization; and (3) spend several hours each week reading stories to people suffering severe loss of hearing or vision. These volunteers routinely express their compassion over a period of time long enough to experience a sense of career in this role.

By contrast casual volunteers engage in much less complex caring activities, which they regard as ‘fun’ or ‘enjoyable’, but which are, like those of the career volunteer, routine and done over a period of time. People who serve meals to the needy, say, once or twice a week throughout the year fall into this category, as do those who, an afternoon a week, solicit donations to charitable, care-giving organizations by way of telephones or manned desks in malls. Some casual hospital volunteers express their compassion as they feed patients who are unable to feed themselves. And people engaged by the Salvation Army to go about the city in trucks picking up used clothing and furniture to give to the poor might well define this activity as (casual) leisure.

Care-based leisure projects also abound. Examples include a one-time period of service with Habitat for Humanity or an international volunteer tourism developmental project such as organized by Youth Challenge International or World-Wide Fund for Nature (some volunteers here return to participate in two or more projects, in which case their leisure begins to evolve into the serious variety, Wearing, 2001). Some disaster volunteers are, in effect, seeking project-based leisure, when they are moved by compassion to help the people suffering from, for instance, the effects of a flood or tornado. Nevertheless, disaster volunteers affiliated with a disaster relief-oriented nonprofit group, such as the Red Cross, who are trained in this specialty and who, on a moment’s notice, are ready to travel to disaster sites, are best viewed as career volunteers (see Britton, 1991).

### 4.1. Encouraging care giving through leisure

Encouraging people to express the compassion they feel for certain other people starts with finding a role through which to do so. Yet, for various reasons too numerous and complex to consider in this article, neither occupational nor personal caring may be options for them. This situation leaves them with the third option, namely, to try one or more of the three types of leisure caring. Explaining this third caring role, in addition to recruiting people to it, requires an understanding of what they are seeking in the way of a leisure experience and its core activities. Additionally effective recruitment of care-giving volunteers hinges on informing potential participants about this special genre of free-time activity.

Disseminating information about leisure activities, in general, and the serious leisure perspective, in particular, is the province of the field of leisure education. Care-oriented volunteer activities are many and varied, but many a would-be volunteer is ignorant of anything approaching the full range of those activities, not to mention their nature. Meanwhile, these people typically know of a fair variety of leisure activities that do interest them, which however, lie beyond the field of care and compassion. In other words, when it comes to activities classifiable as leisure caring, we must recognize that they will have to compete in attractiveness with other appealing free-time activities. Herein lies a major challenge in recruiting compassionate people: encouraging them to follow through on their emotions by educating them about caring activities as described and explained in the serious leisure perspective.

## 5. Conclusion

Thus two important tasks await practitioners in the study of compassion. One is to develop an inventory of care-related leisure activities, with the second being to encourage members of the larger community to pursue them. The serious leisure perspective offers both a framework for creating this inventory and an explanation for why people participate in particular caring activities in their free time.

## Author contributions

The author confirms being the sole contributor of this work and has approved it for publication.

## Conflict of interest

The author declares that the research was conducted in the absence of any commercial or financial relationships that could be construed as a potential conflict of interest.

## Publisher’s note

All claims expressed in this article are solely those of the authors and do not necessarily represent those of their affiliated organizations, or those of the publisher, the editors and the reviewers. Any product that may be evaluated in this article, or claim that may be made by its manufacturer, is not guaranteed or endorsed by the publisher.
